# Signal peptide engineering of a novel M4 family keratinase and the action mechanism in promoting pepper tolerance to salt stress through KerJY23-hydrolyzed feather waste

**DOI:** 10.1016/j.synbio.2026.03.005

**Published:** 2026-03-23

**Authors:** Chao Duan, Yuanxing Wang, Tao Xiong, Zilin Zhang, Huibin Han, Shuaiying Peng

**Affiliations:** a State Key Laboratory of Food Science and Resources, School of Food Science and Technology, Nanchang University, Nanchang, 330047, China; b College of Biological Science and Engineering, Jiangxi Agricultural University, Nanchang, 330045, China; c Jiangxi Province Key Laboratory of Vegetable Cultivation and Utilization, Jiangxi Agricultural University, Nanchang, 330045, China

**Keywords:** Microbial keratinase, Signal peptide library, Feather waste valorization, *Capsicum annuum* L., Transcriptomic analysis, Salt tolerance

## Abstract

Microbial keratinase has attracted significant attention for its ability to convert keratin waste into high-value products with substantial application potential in the agricultural field. However, the low yield and potential pathogenicity of native keratinase producers have significantly hindered their large-scale industrial application. In this study, the novel M4 family keratinase KerJY-23 was heterologous expressed in *Bacillus subtilis*, and a systematic signal peptide engineering was performed. The optimal signal peptide AprE was identified through screening of the signal peptide library, resulting in an extracellular protease activity of 185.37 ± 6.72 U/mg protein, which was 2.17 times that of the native signal peptide. Comparative analysis revealed that signal peptides with amino acid residues ≤5 and a positive charge ≤3 in the N-region, high hydrophobicity in the center of H-region, and a conserved A-X-A motif in the C-region are conducive to the secretory expression of KerJY-23. To further explore the potential agricultural applications of KerJY23-hydrolyzed feather waste, we investigated its effects on salt stress response in pepper (*Capsicum annuum* L.), a commercially significant horticultural crop. Our findings indicated that the exogenous application of feather hydrolysates for 24 h significantly enhanced salt tolerance in pepper plants. Transcriptomic analysis revealed that feather hydrolysates maintain ROS homeostasis and regulate gene expression associated with salt-responsive transcription factors, phospholipid biosynthesis, and plant hormones, ultimately improving pepper tolerance to salt stress. Our work collectively provides a theoretical basis for the utilization of agro-industrial waste in agricultural practices, thereby facilitating its valorization and contributing to sustainable agricultural development.

## Graphical abstract

**Figure:**
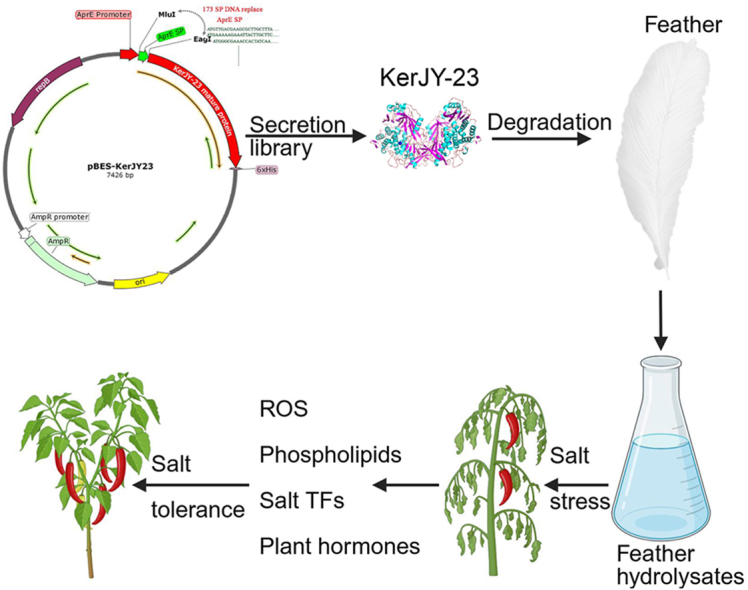

## 1 Introduction

Keratin is a highly cross-linked fibrous protein widely found in biological materials such as animal fur, feathers, claws, and nails. Due to its chemical stability and structural rigidity, the natural degradation of keratin occurs at an extremely slow rate. Feather keratin is a significant source of amino acids, comprising a diverse variety of amino acids, including cysteine, glycine, proline, arginine, and essential amino acids such as valine, leucine, and threonine [1]. However, the presence of intra- and inter-chain cross-linked disulfide bonds, hydrogen bonds, and intermolecular hydrophobic interactions makes feather keratin resistant to conventional proteases [2]. In addition, the increasing demand for chicken meat production has resulted in millions of tons of feather waste accumulation annually, creating substantial waste disposal challenges [3]. Traditional methods for processing feather waste, including incineration, landfilling, and physicochemical treatment, not only contribute to environmental pollution but also result in a severe waste of resources [4].

Keratinase can hydrolyze the rigid and highly cross-linked keratin structure, facilitating the bioconversion of difficult-to-degrade waste into high-value products such as amino acids or bioactive peptides through environmentally friendly processes. These high-value products have been shown to be applicable in agricultural fields, including animal feed and organic fertilizers [5,6]. Microbial keratinases have garnered significant attention for large-scale production due to their ease of synthesis and effectiveness in degrading keratin waste [7]. However, the low enzyme yields and potential pathogenicity of natural producers significantly hinder the industrial application of keratinases. Overexpression of keratinase genes in safe host systems represents an effective strategy to increase keratinase yields and reduce pathogenicity. Given that most keratinases are extracellular enzymes, *Bacillus subtilis* has become the preferred host for heterologous expression of keratinases due to its excellent protein secretion capability [8,9]. Nevertheless, this "simple" approach of overexpressing recombinant keratinases in heterologous hosts still fails to meet the demands of large-scale industrial production.

With the rapid development of genetic and protein engineering technologies, various molecular strategies, for instance, signal peptide optimization, have been employed to effectively enhance the secretory expression yield of recombinant proteins. Compared with promoter engineering and codon optimization, signal peptide is an indispensable element for the secretion of a target protein. Signal peptides are not merely simple address codes for exported proteins; rather, they affect various stages of the entire secretory protein expression process [10]. Therefore, it is evident that the nature of the signal peptide used for the secretion of a desired protein is of crucial importance for the final secretory yield of the specific protein. Research has demonstrated that optimizing signal peptides in most microbial expression systems can significantly improve the secretion efficiency of target proteins. For instance, Xu et al. (2023) reported a 2.3-fold increase in the secretory yield of the thermophilic serine protease TTHA0724 by replacing the *yoaW* signal peptide with the *aprE* signal peptide [11]. Similarly, Dong et al. (2017) achieved an enzyme activity of 473 U/mL by substituting the natural signal peptide of keratinase KerP with the *aprE* signal peptide from *B. subtilis* [12]. The optimal matching of signal peptides with their corresponding mature peptides is crucial for the effective secretory expression of heterologous proteins [13]. However, a specific method or algorithm for designing the optimal signal peptide for target proteins is currently lacking [14,15]. Consequently, constructing a signal peptide library followed by a systematic screening remains a powerful approach to identify the optimal signal peptides for a target protein within a given expression host [10,16].

The hydrolysis of feather keratin by keratinases produces a variety of amino acids that play vital roles in regulating genetic and physiological processes of plants [2,17]. For instance, tryptophan released during feather hydrolysis serves as a precursor for the synthesis of indole-3-acetic acid, a crucial phytohormone that stimulates cell division and elongation, apical dominance, embryogenesis, and root development in plants [18]. Additionally, amino acids also play a pivotal role in plant stress adaptation [[19], [20], [21]]. Salinity represents a crucial abiotic stressor, significantly impeding plant growth and productivity. This is attributed to the increased usage of substandard irrigation water and the consequent soil salinization [22]. Under saline stress, various genes associated with amino acid metabolism exhibit differential expression patterns, underscoring the essential role of amino acids in the salt stress response [23,24]. Pepper (*Capsicum annuum* L.), an important Solanaceae family member, exhibits extensive variability in fruit morphology and enrich flavors [25]. Despite being one of the most extensively cultivated crops globally, pepper is highly susceptible to salinity stress, which markedly hampers its growth and reduces yield [26]. Therefore, if the hydrolysates of feather keratinase can be used to enhance the salt tolerance of peppers in response to salt stress, it would not only replace the plant hormones and organic fertilizers traditionally used in agriculture to cope with salt stress, thus reducing costs, but also address the environmental pollution caused by feather waste disposal. Moreover, to the best of our knowledge, feather keratinase hydrolysates have rarely been used to enhance the salt stress tolerance of peppers.

KerJY-23, a novel keratinase belonging to the M4 metalloprotease family, was previously identified from a highly efficient feather-degrading bacterial strain. Although it was successfully overexpressed in *Escherichia coli*, the protein accumulated as inclusion bodies [27]. Given that KerJY-23 contains a native signal peptide, we explored its secretory expression in *B. subtilis* host in this study. A signal peptide library containing 173 types of signal peptide molecules was constructed for the purpose of discovering the optimal signal peptide for keratinase KerJY-23 which was identified as a novel keratinase in the M4 metalloprotease. Subsequently, a comparative study was performed to reveal the characteristics of signal peptides conducive to secretory expression of KerJY-23 in *B. subtilis* host. Finally, we investigated the potential application of KerJY23-hydrolyzed feather waste in regulating the salt tolerance of pepper plants, as well as revealing its action mechanism. The findings of this work provide a material basis for employing rational or semi-rational strategies to modify signal peptide sequences to further improve the secretory expression level of KerJY-23 in the future, while also expanding the valorization application scope of feather waste through a green and sustainable approach.

## 2 Materials and methods

### 2.1 Strains, plasmids, and materials

The *E. coli* DH5α strain was used for recombinant plasmid preparation, and the *B. subtilis* WB600 strain was used for protein expression. The bacterial genome DNA extraction kit, plasmid extraction kit, Gel DNA extraction kit, and reagents used for PCR amplification were all purchased from Vazyme Biotech Co., Ltd (Nanjing, China). Restriction endonucleases were purchased from New England Biolabs Co., Ltd (Beijing, China). Other chemicals and reagents used in this study were all of analytical grade and commercially available.

### 2.2 Construction of recombinant pBES-kerJY23 plasmid

Primers used for amplifying the target gene of KerJY-23 mature peptide were listed in Table S1. Subsequently, PCR amplification was performed using the genomic DNA of *Bacillus* sp. JY-23 to obtain the target gene. The pBE-S vector was linearized using restriction endonucleases of *Eag*I and *Xba*I, and seamless cloning was conducted by using In-Fusion® Snap Assembly Master Mix purchased from TaKaRa Biotech Co., Ltd (Beijing, China, catalog number: 638947) according to the manufacturer's instructions. The recombinant plasmid was then introduced into the competent *E. coli* DH5α cells, screened through double enzyme digestion of *Eag*I and *Xba*I (Fig. S1), and finally verified by DNA sequencing.

### 2.3 Construction of signal peptide library

Signal peptide library of KerJY-23 was constructed by using *B. Subtilis* Secretory Protein Expression System Kit purchased from TaKaRa Biotech Co., Ltd (Beijing, China, catalog number: 3380) according to the manufacturer's instructions.

### 2.4 Strain screening

#### 2.4.1 Strain cultivation

A single bacterial colony was picked with an inoculating loop and inoculated into 3 mL of sterile LB broth containing 50 μg/mL kanamycin (final concentration), then cultured at 37 °C and 200 rpm for 16 h. Subsequently, the bacterial culture was inoculated into 100 mL of sterile feather-based medium (Chicken feather 4 g/L, K_2_HPO_4_ 1.5 g/L, NaCl 0.3 g/L, MgSO_4_‧7H_2_O 0.025 g/L, CaCl_2_ 0.025 g/L, FeSO_4_‧7H_2_O 0.015 g/L, pH 7.4) with 3% inoculation ratio. Strains were cultured at 37 °C and 200 rpm for 72 h. Feather fermentation broth was obtained and used for subsequent experiments.

#### 2.4.2 Casein hydrolysis circle experiment

A single bacterial colony was picked with a sterile toothpick and inoculated onto a casein agar plate (casein 10 g/L, beef extract 3.0 g/L, NaCl 5.0 g/L, Na_2_HPO_4_ 2.0 g/L, agar 15 g/L, pH 7.4) with five colonies per plate. After incubation at 37 °C for 24 h, the hydrolysis zone diameter of each colony was observed.

#### 2.4.3 Determination of chicken feather degradation rate

The feather fermentation broth was filtered using eight layers of sterile gauze and washed twice with sterile water, and then dried in an oven at 60 °C until constant weight. The weights of the gauzes before and after filtration were measured, respectively, and the feather degradation rate was calculated using the following formula:

$$\text{Feather}\;\text{degradation}\;\text{rate}\;\left(\%\right)=\left(1‐\frac{W1‐W0}{0.4}\right)\times 100\%$$

Where, *W*1 refers to the gauze weight after filtration; *W*0 refers to the gauze weight before filtration.

#### 2.4.4 Determination of extracellular protease activity

The obtained feather fermentation broth was centrifuged at 8000 rpm for 15 min at 4 °C, and the supernatant was then centrifuged again at 10,000 rpm for 20 min at 4 °C. The new supernatant was collected and filtered through a 0.22 μm filter membrane. The filtrate was obtained as feather hydrolysates. The protease activity of feather hydrolysates was determined using Alkali Proteinase (AKP) Activity Assay kit purchased from Solarbio Science & Technology Co., Ltd (Beijing, China, catalog number: BC2305) according to the manufacturer's instructions. One unit of protease activity was defined as the amount of enzyme that catalyzes the hydrolysis to produce 1 μmol of tyrosine per milligram of protein per minute at 40 °C, and expressed as U/mg prot.

#### 2.4.5 Sodium dodecyl sulfate-polyacrylamide gel electrophoresis (SDS-PAGE) analysis

Feather hydrolysates were denatured in 5 × SDS protein loading buffer at 100 °C for 5 min. The electrophoresis process was carried out using 4% (w/v) stacking and 12% (w/v) separating gels in a vertical slab gel apparatus with a voltage of 120 V. The gel was stained with 0.05% Coomassie Brilliant Blue R-250 for 2 h and destained in solutions containing acetic acid, methanol, and water (1:3:6, v/v/v).

### 2.5 Bioinformatic analysis of signal peptides

Online tool SignalP 6.0 (https://services.healthtech.dtu.dk/services/SignalP-6.0/) was used to predict the types, recognition sites, and cleavage probability of signal peptides. Online tool SignalP 4.1 (https://services.healthtech.dtu.dk/services/SignalP-4.1/) was used to predict the D-scores of signal peptides. Amino acid sequence homologous alignment of signal peptides was performed using the ClustalW module in Mega 7.0 software under default settings. Online tool WebLogo (http://weblogo.berkeley.edu/logo.cgi) was used to construct sequence logos from the aligned results of signal peptides.

### 2.6 Free amino acid analysis

Feather hydrolysates of 0 and 72 h were collected and precipitated with 10% sulfosalicylic acid at a 1:1 ratio for over 4 h, then centrifuged at 12,000 rpm for 10 min. The supernatant was collected and filtered through a 0.22 μm filter membrane, and subsequently analyzed by an amino acid analyzer Biochrom 30+ (Biochrom, England).

### 2.7 Plant material and growth conditions

Zunla-1 pepper seeds were surface-sterilized using 0.1% (w/v) potassium permanganate for 15 min and washed with deionized water four to five times. Subsequently, all seeds were germinated in the dark at 28 °C for five days. The germinated seeds were then transplanted into the soil (HAWITA, Shanghai, China, soil/vermiculite, 1:3). The pepper plants were cultivated in a growth chamber at 23 °C under long-day conditions (16 h light/8 h dark), with the light intensity was roughly 200 μmol photons m^−2^ s^−1^ white light.

### 2.8 Transcriptome assay

30-day-old pepper plants were subjected to control (water) and salt stress treatment (300 mM, NaCl), and Salt plus 5 mL of feather hydrolysate (here after referred to as Combine) for 24 h, then the pepper leaves were collected for RNA-seq assay by the Tsingke Biotech Co., Ltd (Beijing, China). Total RNA was extracted using the Super FastPure Cell RNA Isolation kit purchased from Vazyme Biotech Co., Ltd (Nanjing, China, catalog number: RC102-01). Subsequently, libraries were constructed using the VAHTS Universal V6 RNA-seq Library Prep Kit. Transcriptome sequencing and analysis were executed on the Illumina Novaseq 6000 platform (Illumina, California, USA), generating 150 bp paired-end reads. The clean reads were mapped to the recently assembled Zunla-1 genome [28]. Differential expression analysis was carried out with a significance threshold set at q value < 0.05 and fold-change >2 or fold-change <0.5 to identify differentially expressed genes (DEGs). The DEGs were subsequently subjected to the Kyoto Encyclopedia of Genes and Genomes (KEGG) and Gene Ontology (GO) enrichment analyses via ChiPlot online tool (https://www.chiplot.online/). Heatmaps of genes expression were generated via TBtools software (Version 2.3).

### 2.9 Malondialdehyde (MDA) and proline measurements

The MDA quantification kit (COMIN, Suzhou, China, catalog number: MDA-2-Y) and proline assay kit (COMIN, Suzhou, China, catalog number: PRO-2-Y) were used to determine MDA and proline levels, respectively. In brief, 30-day-old pepper plants were exposed to control or salt stress (300 mM, NaCl) for 4 and 24 h with or without feather hydrolysate, respectively. Subsequently, the leaves were harvested and subjected to the MDA and proline assay following the manufacturer's instructions.

### 2.10 ROS assay

The H_2_O_2_ quantification kit (COMIN, Suzhou, China, catalog number: H_2_O_2_-2-Y), CAT enzyme kit (COMIN, Suzhou, China, catalog number: CAT-2-Y), and POD enzyme kit (COMIN, Suzhou, China, catalog number: POD-2-Y) were used to determine H_2_O_2_, CAT, and POD enzyme levels, respectively. In brief, 30-day-old pepper plants were exposed to control or salt stress (300 mM, NaCl) for 4 and 24 h with or without feather hydrolysate, respectively. Subsequently, the leaves were harvested to perform the assay following the manufacturer's instructions.

### 2.11 Quantitative Real-Time PCR (qRT-PCR) assay

1 μg RNA (same RNA from the transcriptome) was used for cDNA synthesis using StartScript II First-strand cDNA Synthesis Kit (GenStar, Beijing, China, catalog number: A224). The qPCRs were performed with 2 RealStar Fast SYBR qPCR Mix (GenStar, Beijing, China, catalog number: A304) using the VQ-100B Real-Time PCR System (Yuanzan, China). The pepper *CaACTIN* gene was used as an internal control, and the relative transcript level was calculated via the 2^−ΔΔCT^ method [29]. Three independent biological replicates were performed, and for each independent biological replicate, the relative transcription level was calculated as the mean of three technical replicates. All qPCR primers used in this study were shown in Table S1.

### 2.12 Statistic analysis

All experiments were performed in triplicate unless otherwise stated. Statistical analysis was performed using multiple paired *t* tests via GraphPad Prism (version 10.5), and data were presented as mean ± standard deviation. ∗*P* < 0.05, ∗∗*P* < 0.01, ∗∗∗*P* < 0.001.

## 3 Results

### 3.1 Construction and screening of signal peptide library for KerJY-23 mature peptide

**Fig. 1:**
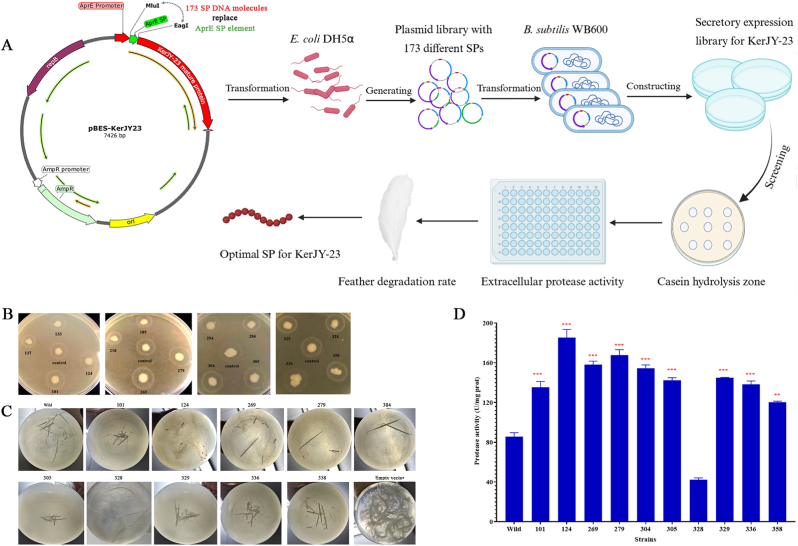
Optimal signal peptide screening for KerJY-23 mature peptide from a secretory expression library. (A) Construction and screening process of signal peptide library; (B) Casein hydrolysis selection via clear zone; (C) Feather utilization efficiency selection via degradation rate; (D) Extracellular protease activity determination. Control: Wild-type strain. ∗∗*P* < 0.01, ∗∗∗*P* < 0.001. ∗∗*P* < 0.01, ∗∗∗*P* < 0.001。

The recombinant plasmid pBES-KerJY23 was used as the initial expression vector for constructing the signal peptide library. A DNA mixture containing 173 different signal peptides derived from *Bacillus subtilis* was inserted into the pBES-KerJY23 plasmid, replacing the native signal peptide on the pBE-S vector (Fig. 1A), thereby creating a secretory signal peptide library consisting of 998 transformants. Subsequently, screening was conducted through casein hydrolysis assays, feather degradation tests, and extracellular protease activity determine to identify the optimal signal peptide for KerJY-23 (Fig. 1A). The screening results showed that strains numbered 101, 124, 269, 279, 304, and 305 exhibited larger hydrolysis zones compared to the wild-type strain (control), which contains the native signal peptide of KerJY-23. In contrast, strains numbered 328, 329, 336, and 358 showed almost no difference when compared to the control (Fig. 1B). Moreover, the feather degradation and extracellular protease activity assays results showed that, all strains had higher feather degradation rates and protease activity than the wild-type strain, except for strain 328 (Fig. 1C). Specially, strain 124 showed the highest extracellular protease activity, reaching 185.37 ± 6.72 U/mg protein, followed by strain 279 (167.70 ± 4.54 U/mg protein) and strain 269 (158.07 ± 2.96 U/mg protein), which were 2.17, 1.96, and 1.85 times higher than the wild-type strain, respectively (Fig. 1D). A distinct overexpressed protein band was observed at 57 kDa, corresponding to the anticipated molecular weight of mature KerJY-23. All selected strains from the signal peptide library exhibited higher secretory expression levels of KerJY-23 compared to the wild-type strain, with the exception of strain 328 (Fig. S2). Notably, the secretory yields of KerJY-23 in strains 101, 124, 269, 279, 304, and 305 were consistently higher than those in strains 328, 329, 336, and 358.

### 3.2 Analysis of the signal peptide information of the selected screened strains

To investigate the differences in the secretory capacity of the selected signal peptides from the secretion library for KerJY-23, the obtained strains were sequenced, and their signal peptides were analyzed and the amino acid sequences of selected signal peptides were presented in Table 1.

**Table 1:** Information on ten selected signal peptides from secretory expression library of KerJY-23.

Strain Number	SP amino acid sequence	SP Name GeneBank Acc. No.	Length	Cleavage site Probability	D-score	Feather degradation rate
101	MKKSIKLYVAVLLLFVVASVPYMHQAALA	Pbp AFG28223.1	29 aa	ALA-AA Sec/SPI 0.972	0.734	85.20 ± 1.60%
124	MRSKKLWISLLFALTLIFTMAFSNMSVQA	AprE AFG28208.1	29 aa	VQA-AA Sec/SPI 0.975	0.720	95.97 ± 0.79%
269	MKKELLASLVLCLSLSPLVSTNEVFA	YjcM AFG28274.1	26 aa	VFA-AA Sec/SPI 0.975	0.830	87.84 ± 1.76%
279	MKKVMLATALFLGLTPAGANA	Pel AFG28199.1	21 aa	ANA-AA Sec/SPI 0.977	0.773	90.58 ± 0.98%
304	MRKSLITLGLASVIGTSSFLIPFTSKTASA	YvcE AFG28249.1	30 aa	ASA-AA Sec/SPI 0.972	0.818	86.55 ± 1.30%
305	MKQGKFSVFLILLLMLTLVVAPKGKAEA	YqgA WP_160221679.1	28 aa	AEA-AA Sec/SPI 0.967	0.741	84.51 ± 1.16%
328	MFAKRFKTSLLPLFAGFLLLFHLVLAGPAAASA	AmyE AFG28243.1	31 aa	AAA-SA Sec/SPI 0.966	0.641	48.10 ± 2.17%
329	MAKPLSKGGILVKKVLIAGAVGTAVLFGTLSSGIPGLPAADA	YncM AFG28189.1	42 aa	ADA-AA Sec/SPI 0.933	0.501	78.27 ± 1.84%
336	MFRLFHNQQKAKTKLKVLLIFQLSVIFSLTAAICLQFSDDTSA	YqxM AFG28254.1	43 aa	TSA-AA Sec/SPI 0.437	0.547	77.58 ± 1.30%
358	MRKKLKWLSFLLGFIILLFLFKYQFSNA	CwlD SPT94302.1	28 aa	SNA-AG Sec/SPI 0.687	0.494	71.46 ± 0.58%
Wild	MKNKRIAMALTAGLTLTMFGAPGAGA		26 aa	AGA-SG Sec/SPI 0.976	0.594	66.99 ± 2.54%

As shown in Table 1, all selected strains exhibited higher feather degradation rates compared to the wild strain (66.99 ± 2.54%), except for strain 328, which had a degradation rate of 48.10 ± 2.17%. Moreover, strains 101, 124, 279, 269, 304, and 305 exhibited superior feather degradation rates, exceeding 80%, compared to strains 328, 329, 336, and 358, which exhibited degradation rates below 80%. Consequently, we categorized the recombinant strains into two groups: the good-performance group (strains of 101, 124, 269, 279, 304, and 305) and the average-performance group (strains of 328, 329, 336, and 358). Among these strains, strain 124 displayed the highest feather degradation rate of 95.97 ± 0.79%, with its signal peptide identified as AprE through protein sequence blast on NCBI online server. The predicted cleavage site of the AprE signal peptide lies between the 28th and 29th amino acid, with a cleavage probability of 0.975. In contrast, strain 328 displayed the lowest feather degradation rate of 48.10 ± 2.17%, and its signal peptide was identified as AmyE, with a predicted cleavage site between the 31st and 32nd amino acids and a cleavage probability of 0.966. Notably, the cleavage probability for the YqxM signal peptide decreased significantly to a value of 0.437. Additionally, the D-score values for signal peptides in the good-performance group were all higher than 0.7, while those in the average-performance group were all below 0.7.

**Fig. 2:**
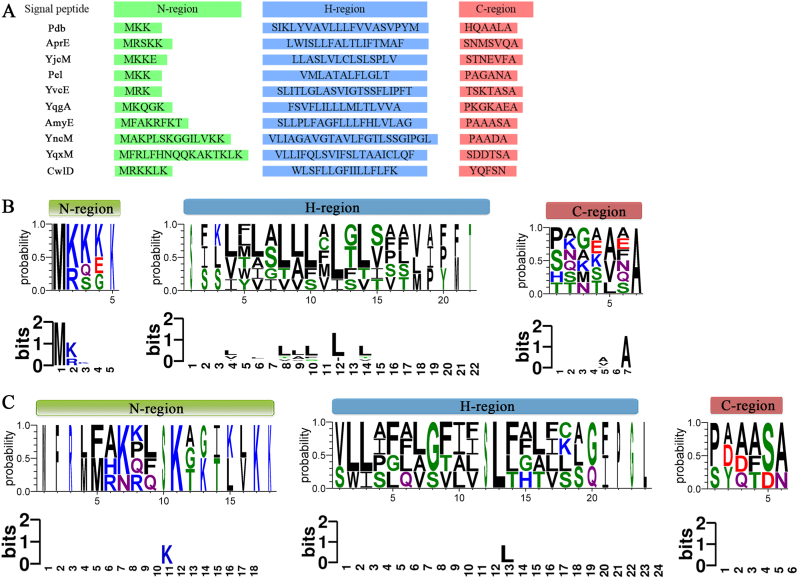
Bioinformatic analysis of N-, H-, and C-regions of 10 signal peptides selected from the secretory expression library for KerJY-23. (A) Amino acid sequences of N-, H-, and C-regions from ten selected signal peptides; (B) Sequence probability (Upper panel) and sequence conservation (lower panel) logos constructed from sequence alignments of the N-, H-, and C-regions of good-performance signal peptides; (C) Sequence probability (Upper panel) and sequence conservation (lower panel) logos constructed from alignments of the N-, H-, and C-regions of normal-performance signal peptides. The colors of amino acid residues in B and C were labeled based on their chemical properties: Black, hydrophobic amino acid (M, L, V, I, F, W, P, A); blue, positively charged amino acid (K, R); red, negatively charged amino acid (D, E); green, polar amino acid (S, G, T, C, Y); purple, neutral amino acid (N, Q).

The results of the homologous alignment of the amino acid sequences in the N-region, H-region, and C-region of good-performance and average-performance signal peptides were shown in Fig. 2. The net positive charge in the N-regions of good-performance signal peptides was generally ≤3, whereas it was ≥3 in signal peptides with average performance. Moreover, the N-regions of good-performance signal peptides typically contained fewer amino acid residues (≤5), in contrast to those of average-performance signal peptides, which exhibited a significant increase in residue length. Notably, the N-regions of YncM and YqxM displayed particularly long residue lengths, with 14 and 16 residues, respectively. In contrast, no significant differences were observed in the amino acid residue number of the H-regions and C-regions between these two signal peptide groups.

Importantly, good-performance signal peptides demonstrated a higher occurrence probability and conservation of the strongly hydrophobic residue leucine (L, colored black) in the center of the H-region (positions between 8 and 14 amino acids) compared to average-performance signal peptides. Additionally, a relative conserved triple L-L-L motif was observed at positions 8 to 10 amino acids in good-performance signal peptides, whereas only one conserved Leu residue was observed at the 13th position in average-performance signal peptides (Fig. 2B and C).

Regarding the C-regions, a conserved alanine (A) was found at the 7th amino acid position, and a relatively conserved A or valine (V) was observed at the 5th amino acid position in the good-performance signal peptides, corresponding to the −3 and −1 position relative to the cleavage site of signal peptidase. In contrast, no conserved amino acids were identified in the C-regions of average-performance signal peptides (Fig. 2B and C).

### 3.3 Free amino acid analysis in KerJY23-hydrolyzed feather waste

**Fig. 3:**
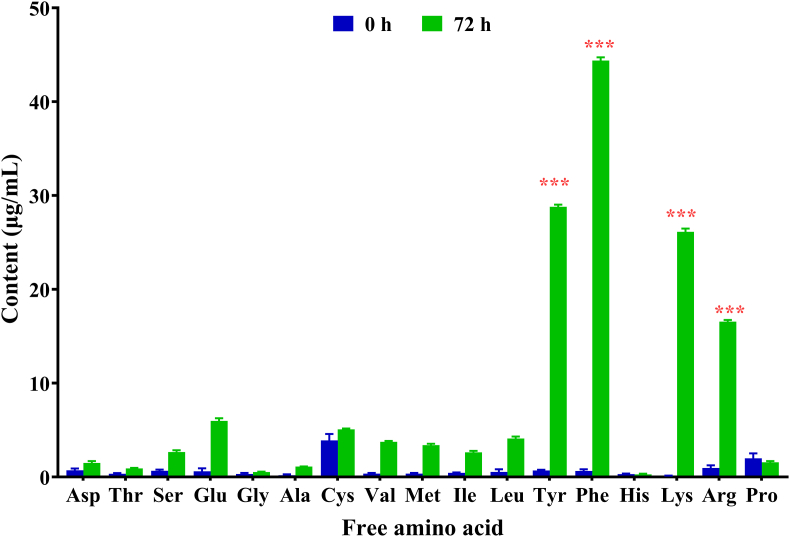
Free amino acid composition and content in feather hydrolysate. ∗∗∗*P* < 0.001. ∗∗∗*P* < 0.001.

Chicken feather hydrolysate was prepared at 0 h and 72 h after inoculation with strain 124. Sterile feather basal medium served as the control (0 h). The composition and concentration of free amino acids in these hydrolysates were subsequently analyzed. As depicted in Fig. 3, a total of 17 free amino acids were identified in the hydrolysates. The concentrations of the majority of these free amino acids increased to varying extents, with the exceptions of histidine and proline, which exhibited decreased concentrations at 72 h. Notably, the concentrations of tyrosine, phenylalanine, lysine, and arginine increased significantly. Among these, phenylalanine was the predominant free amino acid in feather hydrolysates, with a concentration of 44.39 ± 0.34 μg/mL, followed by tyrosine (28.80 ± 0.24 μg/mL), lysine (26.13 ± 0.36 μg/mL), and arginine (16.55 ± 0.18 μg/mL). At 72 h, the concentrations of phenylalanine, tyrosine, lysine, and arginine in feather hydrolysates were 44.44, 68.50, 187.04, and 17.16 times that of 0 h, respectively.

### 3.4 KerJY23-hydrolyzed feather waste improves pepper salt tolerance

Pepper (*Capsicum annuum* L.) is an important horticultural plant with significant economic value. However, it is highly sensitive to salt stress. Amino acids have been shown to regulate the salt stress response in plants. In section 3.3, we identified a spectrum of amino acids in the feather hydrolysate. Therefore, the obtained feather hydrolysate may offer potential benefits in mitigating salt stress in pepper. To investigate this hypothesis, we initially evaluated the effects of applying 5 mL, 20 mL and 50 mL of the hydrolysate on the physiological performance of pepper plants under saline conditions for both 4 and 24-h exposure periods. By analyzing leaf morphology and chlorophyll concentration, we observed that the application of 5 mL of feather hydrolysate effectively enhances salt tolerance of pepper, whereas higher concentrations induced a more pronounced salt-sensitive phenotype (Fig. S3A and B). Consequently, we opted to utilize 5 mL of hydrolysate for subsequent experiments.

**Fig. 4:**
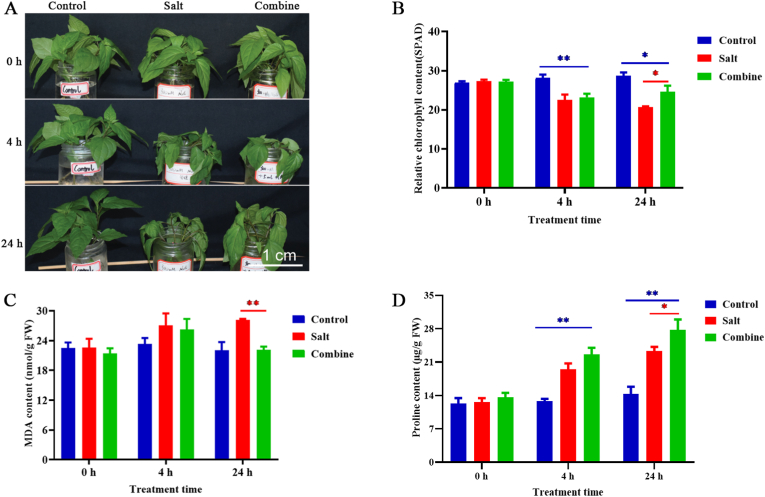
Feather hydrolysate promotes pepper salt tolerance. (A) Phenotypes of 30-day-old pepper plants were subjected to control, salt, and combined (5 mL Feather hydrolysate plus salt) conditions for 0, 4, and 24 h, respectively. Scale bar = 1 cm. (B–-D) Quantification of chlorophyll level (B), MDA content (C), and proline (D) levels in leaves of pepper plants. Control: water treatment, Salt: 300 mM NaCl treatment, Combine: 5 mL feather hydrolysate +300 mM NaCl treatment. ∗P < 0.05, ∗∗P < 0.01.

We exogenously applied 5 mL of feather hydrolysate to 30-day-old pepper plants for 4 h and 24 h, respectively (Fig. 4A). After a 4 h exposure to a 300 mM NaCl solution (salt group), we observed leaf wilting and a decrease in chlorophyll content in the pepper plants (Fig. 4A and B). Compared to the water treatment group (control group), the pepper plants treated with 5 mL of feather hydrolysate combined with 300 mM NaCl (combine group) also exhibited leaf wilting and a significant reduction in chlorophyll levels after 4 h. However, at 24 h, the combined group showed a significant increase in chlorophyll content compared to the salt group. Furthermore, the malondialdehyde (MDA) levels in the leaves of the combine group were significantly lower than those in the salt group at 24 h (Fig. 4C). Additionally, the proline levels in the leaves of the combine group exhibited a notable increase compared to both the control and salt groups at 24 h (Fig. 4D). These results suggest that feather hydrolysate may effectively enhance the stress resilience of valuable horticultural crops, at least in pepper.

### 3.5 Analysis of the physiological mechanisms for enhancing salt tolerance in pepper

#### 3.5.1 KerJY23-hydrolyzed feather waste regulates H_2_O_2_ homeostasis of pepper

**Fig. 5:**
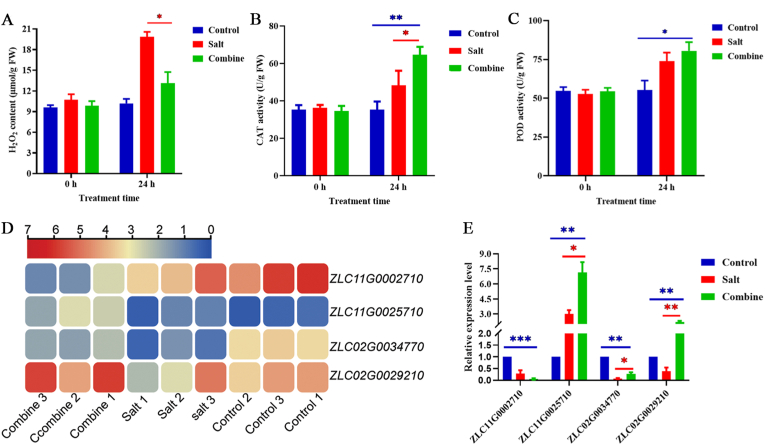
Feather hydrolysate modulates H_2_O_2_ production in leaves of pepper plants under salt stress conditions. Intracellular H_2_O_2_ content (A), CAT activity (B), and POD activity (C) in pepper leaves under different treatments; (D) A heatmap showing the expression patterns of representative H_2_O_2_-related genes with three repetitions from the transcriptomic data. Data are the log2 value of FPKM; (E) qRT-PCR assay of the expression level of ROS-related genes in the leaves of pepper plants under different treatments for 24 h. Control: water treatment, Salt: 300 mM NaCl treatment, Combine: 5 mL feather hydrolysate +300 mM NaCl treatment. ∗*P* < 0.05, ∗∗*P* < 0.01, ∗∗∗*P* < 0.001.

Salinity has been shown to promote H_2_O_2_ production, thus inhibiting plant growth. As shown in Fig. 5A, the H_2_O_2_ content in pepper leaves from the Combine group decreased significantly at 24 h compared to the Salt group, while no significant difference was noted between the Combine and Control groups. After assessing the activity of H_2_O_2_ scavenging enzymes, specifically catalase (CAT) and peroxidase (POD), we observed that the pepper treated with feather hydrolysate under salt stress conditions exhibited a notable increase in CAT and POD activity compared to the Control group at 24 h (Fig. 5B). Relative to the Salt group, CAT activity was significantly elevated in the feather hydrolysate-treated pepper leaves, although no marked difference in POD activity was detected at this time point (Fig. 5C).

Our transcriptomic analysis revealed an obvious downregulation of H_2_O_2_-producing gene *ZLC11G0002710* in the Combine group compared to both the Control group and Salt group. Additionally, genes associated with H_2_O_2_ scavenging, including *ZLC11G0025710* and *ZLC02G0029210*, showed significant upregulation in the Combine group relative to the Salt group at 24 h (Fig. 5D). To validate our transcriptomic results, we performed qRT-PCR to quantify the expression levels of *ZLC11G0002710*, *LC11G0025710*, *ZLC02G0034770*, and *ZLC02G0029210* in pepper leaves under different treatments, which confirmed similar expression patterns of H_2_O_2_-related genes as identified in the transcriptomic data. Specifically, the expression level of *ZLC11G0002710* decreased significantly in the Combine group compared to the Control group. In contrast, the expression levels of *LC11G0025710* and *ZLC02G0029210* increased significantly in the Combine group compared to both the Control and Salt groups (Fig. 5E).

#### 3.5.2 KerJY23-hydrolyzed feather waste modulates phospholipid homeostasis in response to salinity

**Fig. 6:**
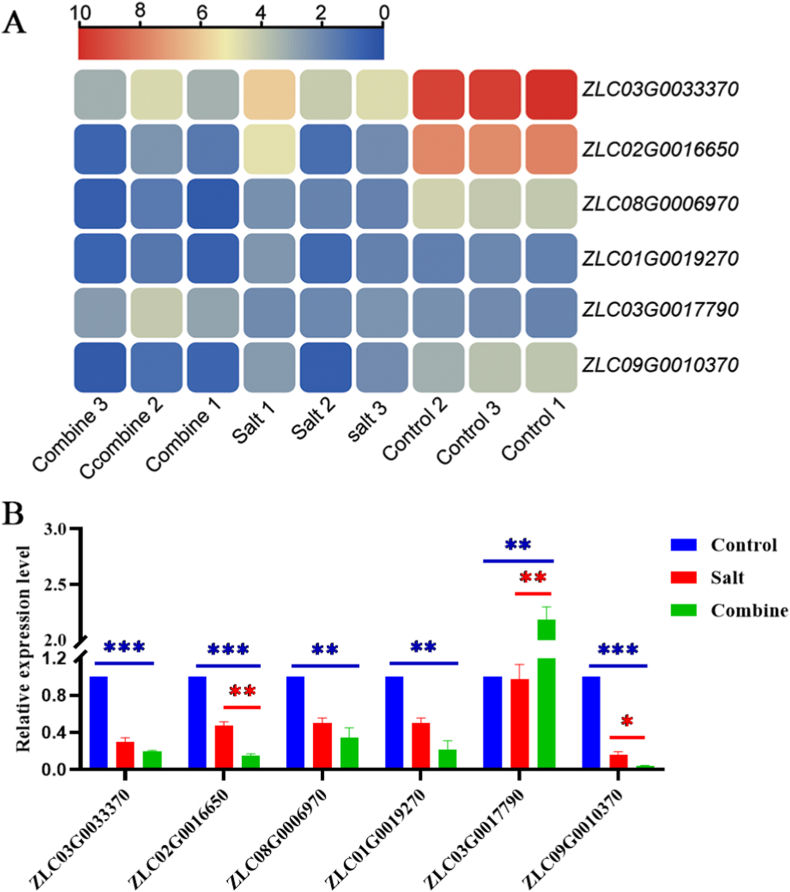
Feather hydrolysate regulates the expression of phospholipid metabolism-related genes in leaves of pepper plants under salt stress conditions. (A) A heatmap showing the expression patterns of phospholipid metabolism-related genes with three repetitions from the transcriptomic data. Data are the log2 value of FPKM. (B) qRT-PCR assay of the expression level of phospholipid metabolism-related genes. Control: water treatment, Salt: 300 mM NaCl treatment, Combine: 5 mL feather hydrolysate +300 mM NaCl treatment. ∗*P* < 0.05, ∗∗*P* < 0.01, ∗∗∗*P* < 0.001.

Our findings indicated that treatment with feather hydrolysate significantly reduced the MDA levels in the leaves of pepper plants under salt stress conditions (Fig. 4C), which serves as a key indicator of lipid peroxidation in cellular membranes. Importantly, several genes involved in phospholipid metabolism were modulated by feather hydrolysate under salt stress conditions (Fig. 6A). We observed a marked downregulation of genes related to lipid-transfer proteins (*ZLC03G0033370*, *ZLC02G00316650*, and *ZLC08G0006970*), phospholipases (*ZLC01G001927*), and inositol polyphosphate 5-phosphatase 4 (*ZLC09G0010370*) in the Combine and Salt group. Moreover, our qRT-PCR analysis further validated the expression pattern of these phospholipid-related genes as identified in transcriptomic data (Fig. 6B). Notably, the expression levels of the lipid-transfer protein-encoding gene *ZLC02G0016650* and *ZLC09G0010370*, which encodes inositol inositol polyphosphate 5-phosphatase 4 involved in the dephosphorylation of lipid molecules, significantly decreased in the Combine group compared to the Salt group (Fig. 6B). Furthermore, the gene *ZLC03G0017790*, which encodes inositol-tetrakisphosphate 1-kinase, exhibited a significant upregulation in the combine group relative to both the salt and control group (Fig. 6A and B). This enzyme is crucial for the biosynthesis of inositol pentakisphosphate, a vital precursor in the formation of biophospholipids. Consequently, our findings propose that the changes in phospholipid composition induced by feather hydrolysate may affect the structure and permeability of cell membranes, thereby improving pepper tolerance to salt stress.

#### 3.5.3 KerJY23-hydrolyzed feather waste regulates multiple signaling pathways in pepper salt stress response

**Fig. 7:**
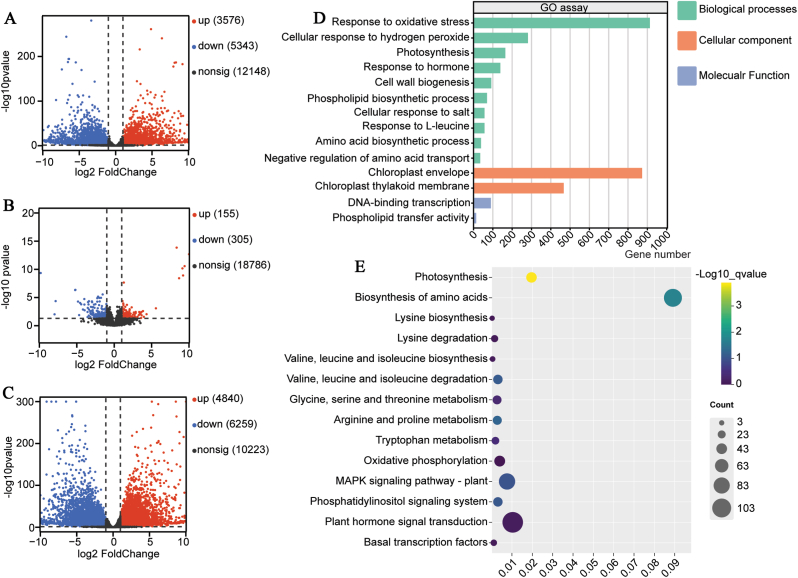
Transcriptome assay in the pepper leaves. The differentially expressed genes in control vs salt group (A), Salt vs combine group (5 mL Feather hydrolysate plus salt) (B), and Control vs combine group (C). GO assay (D) and KEGG assay (E) of the DEGs.

To further elucidate the molecular mechanisms of feather hydrolysate in the promotion of pepper tolerance to salinity, we therefore performed a transcriptomic assay in the leaves of 30-day-old pepper plants treated with Control (water), Salt stress (300 mM NaCl), and Combine (5 mL feather hydrolysate plus salt) for 24 h. The differentially expressed genes (DEGs) were analyzed under different conditions (Fig. 7A, B, and C). In the control vs salt group, 73576 genes were up-regulated and 5343 genes were down-regulated (Supplementary Data S1); 155 up-regulated and 305 down-regulated genes were identified in the Salt vs Combine group (Supplementary Data S2); 4840 up-regulated and 6259 down-regulated genes were identified in the Control vs Combine group (Supplementary Data S3). KEGG and GO analysis further demonstrated that the DEGs were enriched in multiple signaling and developmental processes such as response to salt, photosynthesis, cell membrane biogenesis, amino acid biosynthesis, response to hydrogen peroxide, response to hormone, and transcriptional regulation (Fig. 7D and E). Collectively, our transcriptomic data indicate that feather hydrolysate may regulate gene expression in diverse signaling pathways under salt stress conditions.

Transcription factors (TFs) are proteins that bind selectively to specific DNA sequences to regulate transcriptional activity. Numerous families of TFs, including WRKY, APETALA2 (AP2), ETHYLENE RESPONSE FACTOR (ERF), MYB, and homeobox-leucine zipper family, have been identified as key players in plant responses to salt stress. Our transcriptomic analysis revealed that several salt-responsive TFs were differentially regulated by feather hydrolysate under salt stress conditions. Specifically, the transcript levels of homeobox-leucine zipper protein (*ZLC02G0005020*), WRKY72 (*ZLC11G0018000*), ERF (*ZLC03G0038420*), MYB4 (*ZLC06G0024480*), and WRKY29 (*ZLC01G0002560*) were upregulated in the presence of feather hydrolysate. Conversely, the expression of ERF017 (*ZLC09G0002310*) was downregulated under salinity stress when treated with feather hydrolysate (Fig. S4A). This expression pattern was further validated by our qRT-PCR assay (Fig. S4B). Notably, the expression levels of *ZLC03G0038420* (ERF), *ZLC11G0018000* (WRKY72), and *ZLC06G0024480* (MYB4) were significantly higher in the Combine group than in the Salt group. Whereas, the expression level of *ZLC09G0002310* (ERF017) decreased obviously in the Combine group compared to both the Salt and Control groups. Therefore, the regulation of salt-responsive TFs is a crucial mechanism by which feather hydrolysate enhances salt tolerance in pepper.

It is known that activation of the plant hormones biosynthesis, such as cytokinin, abscisic acid (ABA), auxin, and salicylic acid (SA) or their responsive pathways would promote plant tolerance to salinity. Notably, our transcriptomic data revealed that the expression level of genes related to cytokinin biosynthesis genes (*ZLC10G0022720*, *ZLC02G0009120*) and signaling genes (*ZLC05G0003130*, *ZLC09G0016290*, *ZLC01G0002550*, *ZLC03G0040360*) were upregulated by feather hydrolysate under salt stress conditions (Fig. S5A). In addition, we also found that the transcriptional level of genes associated with abscisic acid signaling (*ZLC12G0005340*, *ZLC01G0026840*, *ZLC02G0014500*, *ZLC08G0013040*, *ZLC08G0013020*) (Fig. S5B), auxin signaling (*ZLC11G0015370*, *ZLC05G0010930*, *ZLC10G0001490*, *ZLC08G0009030*, *ZLC03G0009920*) (Fig. S5C) and SA signaling (*ZLC11G0025710*, *ZLC06G0028030*, *ZLC07G0004540*, *ZLC01G0004100*, *ZLC11G0001150*, *ZLC05G0010930*, *ZLC07G0017460*) (Fig. S5D) was also increased in comparison to the salt stress treatment. Our data hence suggest that feather hydrolysate could activate the plant hormone biosynthesis or signaling pathways, thus alleviating the negative impact of salinity to enable an optimal pepper plant growth.

## 4 Discussion

Microbial keratinases, a class of multifunctional proteases, have garnered increasing attention in the biotechnology field due to their ability to biodegrade recalcitrant keratin waste, thereby facilitating sustainable waste management. Additionally, the bioconversion of keratinous waste into high-value products, such as amino acids and bioactive peptides, contributes to sustainable development by transforming low-cost agro-industrial waste byproducts. However, the commercial application of microbial keratinases is hindered by low enzyme yields and the potential pathogenicity of the native keratinase-producing microorganisms. In this study, we overexpressed a novel M4 family keratinase, KerJY-23, in a generally recognized as safe (GRAS) strain of *B*. *subtilis* to mitigate the potential pathogenicity associated with native producers. Subsequently, we systematically optimized the signal peptide to identify the optimal signal peptide sequence for KerJY-23 to enhance its secretory expression yields. Furthermore, we explored the effects of KerJY23-hydrolyzed feather waste on the salt tolerance of pepper plants and investigated the underlying mechanisms.

By constructing a signal peptide library, we identified the optimal signal peptide, AprE, for the secretory expression of KerJY-23 in *B*. *subtilis* host. The feather degradation rate and extracellular protease activity increased significantly when the native signal peptide of KerJY-23 was replaced with AprE (Fig. 1). To elucidate the characteristics of signal peptide conducive to the secretory expression of KerJY-23, a comparative study was conducted by analyzing two groups of signal peptides: six good-performance signal peptides that effectively enhanced the secretion of KerJY-23, and four average-performance signal peptides that exhibited moderate effects. All ten signal peptides belong to the Sec/SPI category, characterized by a conserved three-region structure: a positively charged amino terminus (N-region), a central hydrophobic core (H-region), and a polar carboxyl terminus (C-region).

It's note worthing that the effect of increasing the net positive charge of the N-region on protein secretion remains controversial [30]. While some studies suggest that a higher net positive charge enhances secretion efficiency, others indicate that the optimal charge may vary depending on the target protein and host system. For instance, one study reported that point mutations increasing the net positive charge of the N-region to +4 resulted in a marked reduction in chimeric lichenase secretion [31]. Similarly, increasing the net positive charge of the *Streptomyces tendae* α-amylase inhibitor tendamistat signal peptide from +2/+3 to +4/+6 adversely affected tendamistat secretion [32]. In line with these observations, our study demonstrated that a net positive charge of ≤3 in the N-region benefits KerJY-23 secretion in *B. subtilis*, supporting the view that a higher net positive charge in the signal peptide N-region is not universally advantageous or necessary for efficient protein secretion. Additionally, good-performance signal peptides generally possess shorter N-regions (≤5 amino acids), whereas average-performance signal peptides exhibit significantly longer N-regions. Notably, YncM and YqxM signal peptides contain 14 and 16 residues in the N-regions, respectively (Fig. 2A), which far exceed the typical length of 5 residues in N-regions [33]. In this study, we demonstrated that N-region composed of five or fewer amino acids facilitates the secretion of KerJY-23, which aligns with the findings of Eom et al., who reported that a short N-region length enhances the secretion yield of chitosanase. Specifically, a naturally occurring mutant signal peptide, characterized by a six-amino-acid deletion in the N-region relative to the wild-type, lead to a 2.37-fold increase chitosanase serection [34].The relative contributions of the net positive charge and the length of the N-regions to the secretory expression efficacy of KerJY-23 remain to be elucidated through further investigation. The H-region, which constitutes the hydrophobic core functional area of the signal peptide, typically comprises approximately 7 to 15 amino acids, predominantly including leucine, isoleucine, and valine residues [35]. The hydrophobicity of amino acids within the H-region is correlated with translocation efficiency. It has been reported that mutating the glycine at position 25 in the H-region of the TorA signal peptide to a more hydrophobic amino acid can significantly enhance the translocation efficiency of the precursor protein [36]. In this study, the occurrence and conservation of strongly hydrophobic residues, especially leucine, in the H-region of good-performance signal peptides were significantly higher than those in the average-performance signal peptides (Fig. 2B and C). This finding aligns with a previous report indicating that an increase in leucine residues in the H-region of the *E*. *coli* alkaline phosphatase signal peptide can markedly improve precursor translocation efficiency [37]. Furthermore, the H-region exhibits a specific arrangement preference for hydrophobic residues. It has been shown that the presence of strongly hydrophobic residues in the center of the H-region or near the C-region can significantly enhance the secretion of the target protein [38]. Our results further corroborate this finding: good-performance signal peptides exhibited a higher probability of Leu occurrence at the center of the H-region, specifically between amino acid positions 8 and 14, and featured a relatively conserved triple Leu motif at positions 8 to 10. In contrast, signal peptides with average performance primarily displayed a relatively conserved Leu residue solely at position 13 within the H-region (Fig. 2B and C). Therefore, we conclude that the presence of strongly hydrophobic amino acids, particularly leucine, in the center of the H-region can effectively promote the secretory expression of KerJY-23 in *B. subtilis*. The C-region is composed of approximately 3 to 7 neutral or polar amino acids and contains the cleavage site for signal peptidase (SPase). Key residues in the C-region are located at positions −3 and −1 relative to the cleavage site, which exhibit a conserved A-X-A motif, commonly referred to as the (−3, −1) rule [30]. Alanine is preferred at both −3 and −1 positions, with the presence of alanine at −1 position being crucial for the correct recognition and cleavage by SPase [33]. In this study, the C-region of high-performance signal peptides generally contains a conserved alanine at −1 position (the 7th amino acid residue) and either an alanine or valine residue at −3 position (the 5th amino acid residue). In contrast, signal peptides with average performance did not exhibit any conserved amino acids in this region (Fig. 2B and C). Therefore, we speculate that the increased secretion efficiency of KerJY-23 may be related to the conserved alanine at position −1 and alanine or valine at position −3 in the C-region of the signal peptide. These residues conform to the A-X-A rule, facilitating effective recognition and cleavage by SPase, thereby enhancing the secretion efficiency of KerJY-23. This finding is consistent with the study by Xue et al., which demonstrates that signal peptides featuring A-X-A or V-X-A motifs exhibited higher secretion efficiency in the secretory expression of α-amylase in *Saccharomyces cerevisiae* [39].

Amino acids play a pivotal role in plant growth and stress resistance, such as salinity tolerance studies [[19], [20], [21]]. We identified 17 free amino acids in the KerJY23-hydrolyzed feather hydrolysate. Among these amino acids, tryosine (Tyr), phenylalanine (Phe), lysine (Lys), and arginine (Arg) were identified as the predominant amino acids (Fig. 3). This discovery led us to investigate the agricultural application of the high-value products of feather waste. We therefore assessed the effects of feather hydrolysate on the salinity stress response in pepper plants. Our findings demonstrate that the exogenous application of 5 mL of feather hydrolysate substantially improves the salinity tolerance of pepper plants (Fig. 4). To uncover the underlying regulatory mechanisms, we performed a transcriptomic assay. Our results indicated that feather hydrolysate regulated the expression of genes related to H_2_O_2_, salt-responsive TFs, phospholipid metabolism, and hormonal pathways under saline conditions (Fig. 7). H_2_O_2_ homeostasis in plants is intricately regulated by the equilibrium between production and scavenging enzymes. We further showed that feather hydrolysate modulated the levels of H_2_O_2_-associated enzymes (Fig. 5). Numerous studies have shown that H_2_O_2_ is also involved in plant hormone pathways under salt stress conditions [40,41]. A couple of genes related to plant hormone signaling pathways, including cytokinin, auxin, abscisic acid, and salicylic acid, were upregulated by feather hydrolysate under salt stress (Fig. S5), potentially linking the H_2_O_2_ and plant hormonal signaling. Moreover, H_2_O_2_ is involved in lipid oxidation [42], leading to compromised cell membrane permeability. Phospholipids also regulate H_2_O_2_ signaling in stress adaptations [43]. Genes related to phospholipid metabolism were modulated by feather hydrolysate (Fig. 6), suggesting a potential link to H_2_O_2_ signaling with phospholipids. Nonetheless, the interplay between H_2_O_2_, signaling, phospholipids, and plant hormones in feather hydrolysate-mediated salt tolerance requires further investigation. Notably, future studies involving mutants associated with the identified DEGs are anticipated to provide novel insights into the biological functions of these DEGs in the salinity response mediated by feather hydrolysate. Additionally, plant hormones, including abscisic acid, auxin, cytokinins, and salicylic acid, are essential for plant adaptation to environmental stimuli [[44], [45], [46]]. WRKY and ERF transcription factors are known to regulate hormone signaling in response to salt stress [44,47,48]. We discovered that *ZLC11G0018000*, *ZLC02G005020*, *ZLC01G0002560*, *ZLC09G0002310*, and *ZLC03G0038420*, encoding WRKY and ERF homologs, were regulated by feather hydrolysate (Fig. S4), suggesting that feather hydrolysate may also modulate plant hormonal pathways via the regulation of salt-responsive TFs in the pepper salt stress response. In addition, TFs also play a central role in regulation of ROS signaling in stress adaptions [49], this may suggest the interplay between ROS, TF, and plant hormones. Nevertheless, the interaction of phytohormones, salt-responsive TFs, plant cell wall, phospholipids, and amino acids in feather hydrolysate-mediated salinity response necessitates further investigation. Notably, future studies involving mutants associated with the identified DEGs are anticipated to provide novel insights into the biological functions of these DEGs in the salinity response mediated by feather hydrolysate. It is also particularly noteworthy to investigate the impact of feather hydrolysate on other environmental stresses, such as drought, temperature, and abiotic factors across a range of crops and horticultural species. Furthermore, small signaling peptides have been show to play a role in salt stress response, this may indicate that keratinase-derived peptides may also play a role in pepper salt stress response, but this requires further investigations.

## 5 Conclusion

In summary, we successfully screened for the optimal signal peptide AprE from a library of 173 distinct signal peptide molecules derived from *Bacillus subtilis*, which significantly enhanced feather degradation efficiency and extracellular protease activity. Further comparative analysis of signal peptides revealed that those beneficial for KerJY-23 expression possessed characteristics such as an N-region with no more than five amino acids, a net positive charge not exceeding three, a hydrophobic H-region with high hydrophobicity at its center, and a conserved A-X-A motif in the C-region. Subsequent studies demonstrated that KerJY23-hydrolyzed feather waste enhanced salt tolerance in pepper (*Capsicum annuum* L.) by modulating H_2_O_2_ homeostasis and regulating the expression of genes involved in phospholipid biosynthesis, salt-responsive TFs, and plant hormones. These findings provide a foundation for future strategies to further improve the secretory expression of keratinase and highlight the potential of microbial keratinase to convert agricultural industrial waste into high-value products through a green and sustainable approach, offering a theoretical basis for its application in agricultural practices.

## CRediT authorship contribution statement

**Chao Duan:** Writing – original draft, Investigation, Conceptualization. **Yuanxing Wang:** Software, Data curation, Conceptualization. **Tao Xiong:** Methodology, Formal analysis. **Zilin Zhang:** Writing – review & editing, Software. **Huibin Han:** Project administration, Funding acquisition. **Shuaiying Peng:** Writing – review & editing, Software, Resources, Project administration, Funding acquisition.

## Funding

This work was supported by a grant from the 10.13039/501100001809National Natural Science Foundation of China (Grant number: 32360230), and 10.13039/501100004479Jiangxi Provincial Natural Science Foundation (Grant number: 20252BAC240626).

## Declaration of competing interest

The authors declare that they have no known competing financial interests or personal relationships that could have appeared to influence the work reported in this paper.
