# Supplementary material for: Signal peptide engineering of a novel M4 family keratinase and the action mechanism in promoting pepper tolerance to salt stress through KerJY23-hydrolyzed feather waste
Source: Synth Syst Biotechnol. 2026 Mar 23;13:480–92. doi: 10.1016/j.synbio.2026.03.005 (PMC13049534; doi:10.1016/j.synbio.2026.03.005)
Supplement: Multimedia component 1 [file mmc1.docx]

# Supplementary material for

**Signal peptide engineering of a novel M4 family keratinase and the action mechanism in promoting pepper tolerance to salt stress through KerJY23-hydrolyzed feather waste**

Chao Duan^1#^, Tao Xiong^1#^, Yuanxing Wang^1*^, Zilin Zhang^2^, Huibin Han^2^^, 3, *^, Shuaiying Peng^2, *^

*^1^* *State Key Laboratory of Food Science and Resources, School of Food Science and Technology, Nanchang University, Nanchang 330047, China*

*^2^ College of Biological Science and Engineering, Jiangxi Agricultural University, Nanchang 330045, China*

*^3^ Jiangxi Province Key Laboratory of Vegetable Cultivation and Utilization, Jiangxi Agricultural University, Nanchang 330045, China.*

* **Corresponding author:** Shuaiying Peng, Yuanxin Wang, Huibin Han

E-mail: [sypeng@jxau.edu,cn](mailto:sypeng@jxau.edu,cn) (Shuaiying Peng)

E-mail: [yuanxingwang@ncu.edu.cn](mailto:yuanxingwang@ncu.edu.cn) (Yuanxing Wang)

E-mail: [huibinhan@jxau.edu.cn](mailto:huibinhan@jxau.edu.cn) (Huibin Han)

^#^ These authors contributed equally.


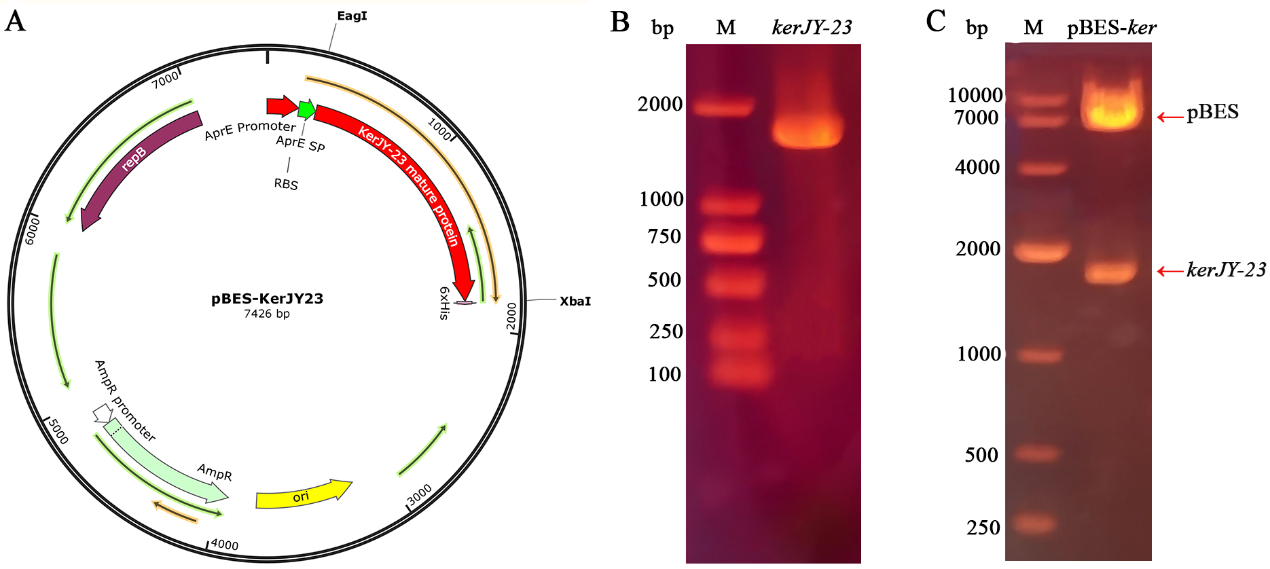


**Fig.S1** Recombinant plasmid construction of pBES-KerJY23. (A): Molecular map of recombinant plasmid pBES-KerJY23; (B): PCR amplification result of kerJY-23 gene without signal peptide sequence; B: Double endonucleases digestion result of the recombinant plasmid. M: DNA marker.


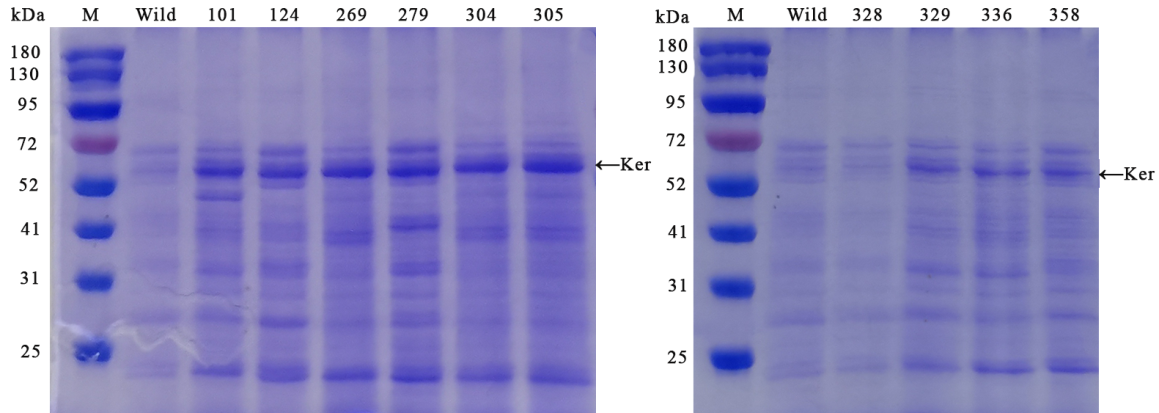


**Fig. S2** SDS-PAGE analysis of the extracellular expression level of KerJY-23.


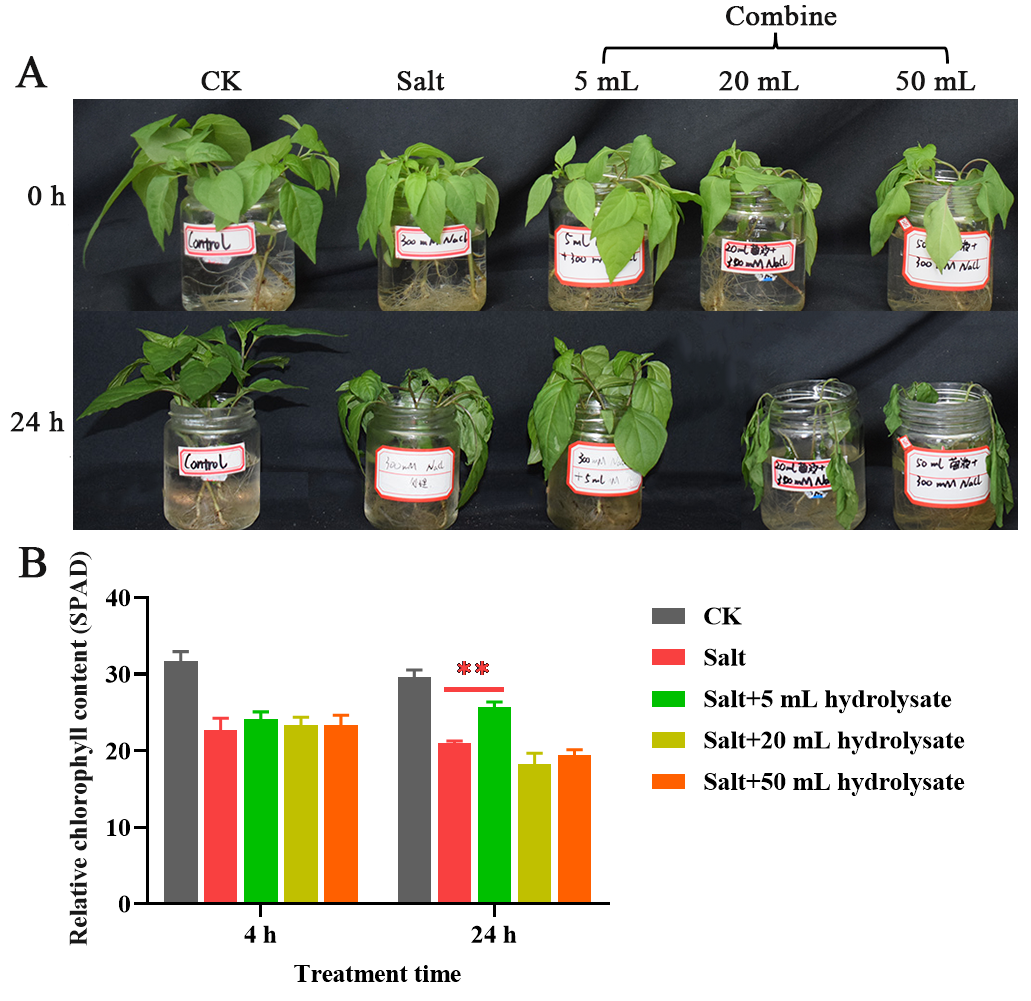


**Fig. S3** The effects of different feather hydrolysate treatment on salt tolerance of pepper. (A): Leaf morphology; (B): Relative chlorophyll content.


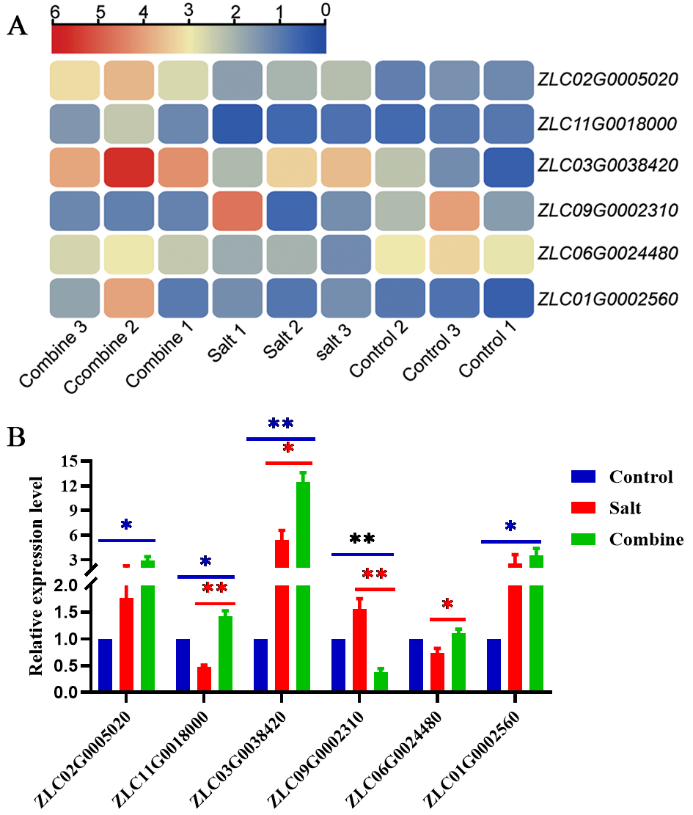


**Fig. S4** Feather hydrolysate regulates the expression of salt-responsive transcription factors in leaves of pepper plants under salt stress conditions. (A): A heatmap showing the expression patterns of salt-responsive transcription factors with three repetitions from the transcriptomic data. Data are the log2 value of FPKM. (B): qRT-PCR assay of the expression level of salt-responsive transcription factors. Control: water treatment, Salt: 300 mM NaCl treatment, Combine: 5 mL feather hydrolysate + 300 mM NaCl treatment. * *P* < 0.05, ** *P* < 0.01


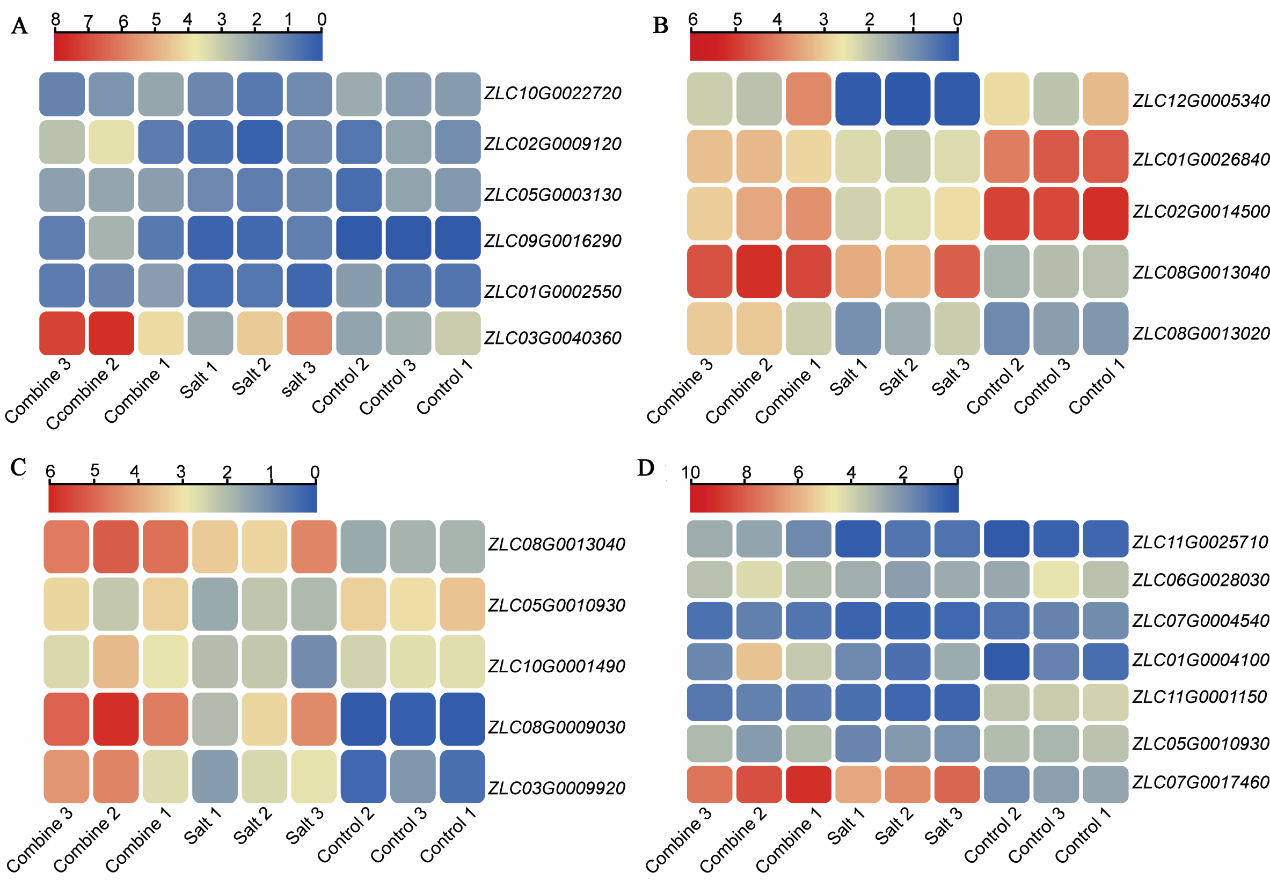


**Fig. S5** Feather hydrolysate regulates the expression of plant hormone pathway genes in leaves of pepper plants under salt stress conditions. A heatmap showing the expression patterns of cytokinin biosynthesis and signaling genes (A), ABA signaling (B), auxin signaling (c) and SA signaling (d) related genes with three repetitions from the transcriptomic data. Data are the log2 value of FPKM. The color scale shows their expression level as indicated at top.

**Table S1.** **Primers used in this study.**

| **Primer Name** | **Forward（5′-3′）** | **Reverse（5′-3′）** |
| --- | --- | --- |
| *kerJY-23* | GCGCAGGCTGCGGCCGGTGCGGATGTAAAGAATGTACTTTC | GATGGTGATGTCTAGAATATACTCCAACTGCATCAAAGG |
| *CaACTIN* | GACGTGACCTAACTGATAACCTGAT | CTCTCAGCACCAATGGTAATAACTT |
| *ZLC11G0002710* | GGCGTGGCTGAAACAACAAT | AGTCAAACCAACAACACCGC |
| *ZLC11G0025710* | ACTAAAAGGCAGCGGTGAAG | CGGCCAATCTAACTGGCGA |
| *ZLC02G0034770* | CCCACTTTATCACAGCGCCA | CGGTGGTGGGTGAAGTGATT |
| *ZLC02G0029210* | TGTGCTGACATTCTCGCCTT | GCAGCTTTCCTTCCACGTTC |
| *ZLC03G0033370* | GCCAAGCTACCACAACTCCT | GAGCAGTCAATGGAAGGGCT |
| *ZLC02G0016650* | TAACCTGTGGCGAAGTCACG | GGCGTTCGCTAGTCTCCTAA |
| *ZLC08G0006970* | AAACGTGCCTTCAGAGTGCT | TTGAGGCAACGCTCTTCACA |
| *ZLC01G0019270* | GGCACAGTTGTTCATGGTCC | AGAGTCAGTTCTGGGCTCCT |
| *ZLC03G0017790* | GAGCGATGAGAGCTTAGCCA | CAGGCACCTTAGCATAGCCA |
| *ZLC09G0010370* | GAACCCCTGCTTGGTGTGAT | TGATGGCGGTTGATGGACTC |
| *ZLC02G0005020* | TGCGCGACACTAACAGATGA | TACGTTGAGGATGGCTGCTG |
| *ZLC11G0018000* | TCGAGGCAACATCTCTGAAC | AGCATCCCCATAGTCGTCAG |
| *ZLC03G0038420* | TGACGACTCCGAAGAGATGC | CTTGGCTGACAACCTCGGAT |
| *ZLC09G0002310* | GCTGCTAGGGCTTTTGATGC | GCCGGAGTCATCAACCTACC |
| *ZLC06G0024480* | GATGGGGTTGAAGAAAGGGC | GCAAGTTTAGGAAGGGCACG |
| *ZLC01G0002560* | AACAACAAGTGGTGGCTGCT | ACACCCCTTTGAGCTACTGC |
